# Beyond genetics: can micro and nanoplastics induce epigenetic and gene-expression modifications?

**DOI:** 10.3389/freae.2023.1241583

**Published:** 2023-08-21

**Authors:** Anna M. G. Poma, Patrizia Morciano, Massimo Aloisi

**Affiliations:** ^1^ Department of Life Health and Environmental Sciences, University of L’Aquila, L’Aquila, Italy; ^2^ INFN—Laboratori Nazionali Del Gran Sasso, L’Aquila, Italy

**Keywords:** microplastics, nanoplastics (NPs), epigenome, *in vivo* effects, plants and animals

## Abstract

Plastic pollution is becoming a worldwide crisis. It can be found in all environmental matrices, from the seas to the oceans, from dry land to the air we breathe. Because of the various types of plastic polymers and waste degradation methods, the types of plastic particles we are exposed to are quite diverse. Plants and animals are continuously exposed to them, and as the top of the food chain, humans are as well. There are numerous studies that confirm the toxicity of these contaminants, yet there is still a significant vacuum in their epigenetics effects and gene expression modifications. Here we collect studies published to date on the epigenetics effects and gene expression modulation induced by micro and nanoplastics. Although published data are still scarce, it is becoming evident that micro- and nanoplastics, whether acutely or chronically administered, do indeed cause such changes in various model organisms. A future challenge is represented by continuing and deepening these studies to better define the molecular mechanisms underlying the observed toxic effects and above all to translate these results to humans to understand their impact on health.

## Introduction

Although plastic pollution has only recently achieved media notoriety, in the scientific literature, the negative impact of this environmental contamination on living organisms has been known since 1969, when two scientists first described the effects on seabirds of plastic fragments (Kenyon et al., 1969). In the paper, the authors found in more than 70 out of 100 Albatrosses about 2 g of plastic residues identifying toys, pouches and caps. The researchers hypothesized unintentional ingestion by the Albatrosses while fishing and consequently death from digestive and airway obstruction. By 2023, about 170 trillion plastic residues were estimated to be floating in the seas and oceans (Eriksen et al., 2023) subjected to transport by currents that concentrate them in areas of higher density, which can lead to the formation of large plastic islands varying greatly in concentrations in different areas of the Planet (Huserbråten et al., 2022).

Today, a term called “plasticosis” has been coined investigating the sequential effects, mainly fibrosis, that occur in seabirds due to ingestion of plastics (Charlton-Howard et al., 2023). The Shearwaters (*Ardenna carneipes*), collected dead, were analyzed for plastic ingestion and for plastic-induced fibrosis in the proventriculus by histopathological analysis. On a scale with grade zero no effect, going up one begins to observe progressive thinning of the collagen layer and increased disorganization of the submucosa and tubular glands of the proventriculus until the last grade, the fifth, with a total loss of the structure of the tubular glands and total disorganization of the submucosa. The authors concluded that the extent and severe presence of fibrosis is a consequence of plastic ingestion. It would be interesting to extend the study to other animal models to see if the term “plasticosis” can also be used more extensively on other organisms.

Once plastic waste is dispersed into the environment, it undergoes the action of chemical and physical processes that lead to degradation with the formation of fragments of various sizes (Figure 1A) (Lambert et al., 2016a; Lambert et al., 2016b). Degradation mechanisms are triggered by different factors, e.g., salinity, UV, physical interactions, and oxidation and generally lead to the formation of chains of radical reactions with release of small fragments (Gewert et al., 2015). Recently, an interesting publication indicates also biological factors, such as the ingestion of microplastics in crustaceans, as promoters of the degradation process (Dawson et al., 2018). Nowadays, there is still no legislative classification for plastic pollution, therefore the scientific community follows the general classification assigned to micro- and nanomaterials. Specifically, nanoplastics (NPs) include fragments of size between 1 and 100 nm, microplastics (MPs) between 101 μm and 5 mm and macroplastics if larger. Sometimes it is also included the term mesoplastics to refer to those between 1 and 5 mm (Ng et al., 2018). To follow the most recent publications on marine concentrations of MPs, a database has recently been developed that illustrates the results directly on a global map and shows how concentrations vary worldwide (Čerkasova et al., 2023). For NPs, there are still no widespread technologies for routine capillary sampling other than complex systems that, however, belong to only a few laboratories (Cerasa et al., 2021; Mariano et al., 2021). Although many cited works refer to aquatic matrices and organisms, plastic pollution is also a widespread phenomenon on lands (Hu et al., 2022). Even the air we breathe can contain traces of plastic fragments formed by aerosolization of polluted water or from emissions from motor vehicles or industrial processes in a wide variety of shapes and sizes (Abbasi et al., 2019; Allen et al., 2019; Bhat et al., 2023). In addition, the progressive accumulation of plastic waste can be used by geologists to identify our Era, as a stratigraphic indicator (Zalasiewicz et al., 2016).

**FIGURE 1:**
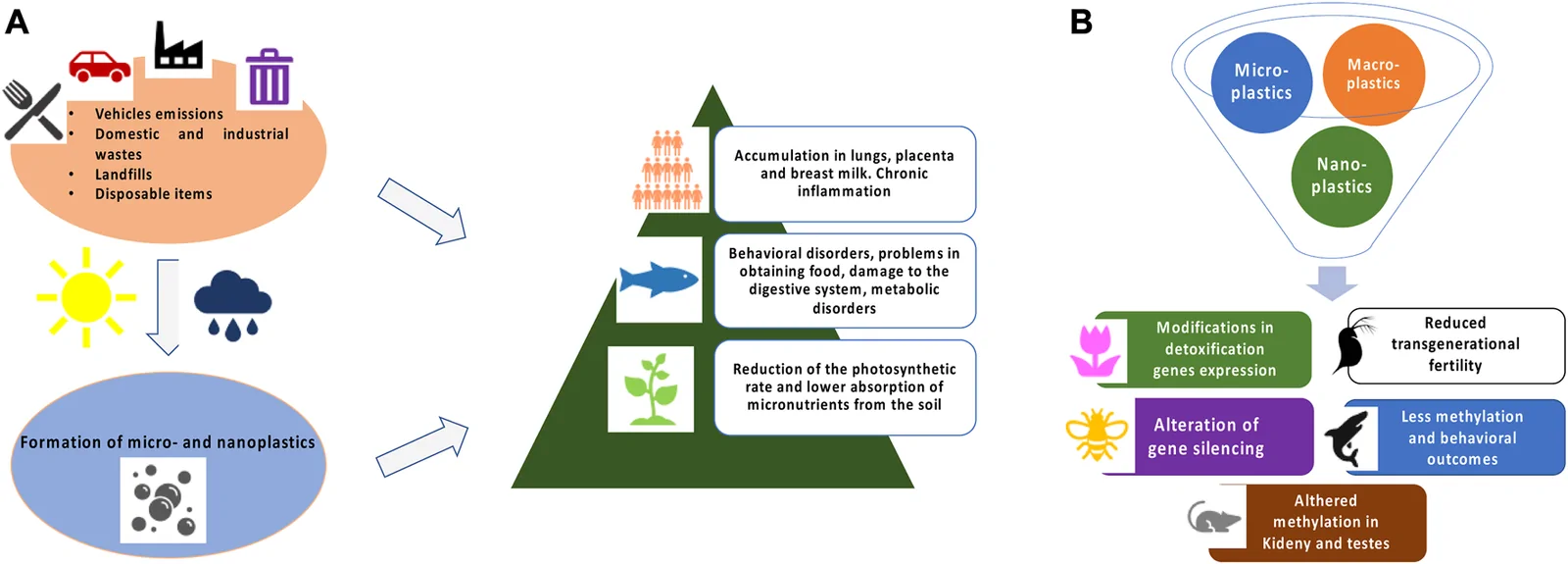
**(A)** Schematic representation of MPs and NPs formation and general toxicity in exposed living organisms. **(B)** Brief summary of epigenetic and gene expression modifications in different model systems.

The growing need to understand the effects of plastic pollution and to standardise knowledge of their impact on our health makes it essential to continue and deepen studies on this global environmental problem.

The topic of this mini-review is to collect published literature on the epigenetic alterations and gene expression modulation induced by MPs and NPs.

### General effects

The adverse effects induced by MPs and NPs were recently summarized by the World Health Organization (WHO) report manifesting the need to define the risk to which humans have been exposed for decades (WHO, 2022). Many studies have been carried out *in vitro* and reported a reduced cell viability likely caused by oxidative stress with increase in Reactive Oxygen Species (ROS) and detoxifying enzymes such as catalase, superoxide dismutase 1 and 2, and glutathione peroxidase. Increased levels of inflammation markers such as interleukins and TGF β were also observed (Hwang et al., 2019; Rubio et al., 2020). This stress in turn led to genotoxic damage assessed by comet and micronuclei assays (references in Table 1). In order to investigate the effects on organism level, several *in vivo* studies have been performed that show that MPs and NPs accumulate mainly in liver and fatty tissues indicating that they cross tissue barriers reaching different organs. The effects observed *in vivo* follow similar trends to those on cells (references in Table 1). More specific effects on complex organism’s aspects such as behavior, immune response, nervous system and microbiota, just to mention a few, were also observed.

**TABLE 1:** Summarizes *in vitro* and *in vivo* general effects and related references.

Model	Particles size	Outcomes	References
*In vitro* studies			
Intestinal cells	20 nm–10 μm	Reduced cell viability and intestinal barrier integrity, inflammation, genotoxicity, oxidative stress, apoptosis, alteration of the cell cycle	Lehner et al. 2020, Stock et al. 2019, Liu et al. 2020, Yan et al. 2020
			Vecchiotti et al. 2021
Lung cells	60 nm–2 μm	Reduced cells viability, ROS formation, inflammation, genotoxicity	Lim et al. 2019, Xu et al. 2019, Paget et al. 2015
Hepatic Cells	15 nm–200 nm	Reduced cells viability, ROS formation, genotoxicity proliferation, hemolysis	Johnston et al. 2010, Kawata et al. 2019
Nervous system cells	40 nm–10 μm	Reduced cells viability, ROS formation, inflammation	Schirinzi et al.2017
			Raghnaill et al. 2014
Epithelial cells	100 nm–200 μm	Reduced cells viability, ROS formation, genotoxicity	Choi et al. 2020, Poma et al. 2019
*In vivo* studies			
*Drosophila melanogaster*	10 nm–800 μm	Oxidative stress, increase in expression of stress response genes, genotoxicity	Alaraby et al. 2023
*Danio rerio*	50 nm–45 μm	Behavior alterations, Oxidative stress, less viability, movement reduction	Brandts et al. 2020, Chen et al. 2017
Mouse	25 nm–5 μm	Oxidative stress, neurotoxicity, metabolic disorder, intestinal barrier dysfunction, dysbiosis	Deng et al. 2018, Jin et al. 2018, Rafiee et al. 2018
Plants and algae	60 nm–1,000 μm	Reduced micronutrients uptake, photosynthetic rate, chromosomes aberrations	Karalija et al. 2022 Gopinath et al. 2019

### Epigenetics and gene expression modulation

Epigenetics studies heritable effects that do not involve mutations in the DNA sequence. Epigenetics is closely linked to developmental processes and explains how an identical set of genomic instructions can give rise to the plethora of cells in a single organism. Epigenetics, turning on and off the expression of certain genes, drives the developmental processes controlling cells and tissues differentiation (Holiday, 2006). Moreover, epigenetics, through molecular processes, determines the plasticity of living organisms towards their environment allowing them to respond to many different external cues and stimuli. The first epigenetic mechanisms discovered are DNA methylation and histone modifications (Li, 2021). DNA methylation is still the analysis most frequently applied as well as the first to be historically characterized (Mattei et al., 2022). Histone modifications are more complex since not only there are different types of histones, but they can go through many types of modifications, e.g., methylation, phosphorylation and acetylation (Stillman, 2018).

Environmental epigenetics deals with the study of epigenetic alterations caused by environmental factors (Bollati and Baccarelli, 2010). For instance, it has been seen how some crustacean species respond to rising water temperatures by modulating genes involved in temperature stress response demethylating and acetylating histones (Hofmann, 2017; Eirin Lopez and Putnam, 2019). Several studies have proved that environmental pollutants can induce epigenetic modifications. A well-known example is the arsenic that induces several pathologies like cardiovascular diseases and cancer caused by general DNA hypomethylation due to arsenic detoxification pathway (Arita and Costa, 2009; Domingo-Relloso et al., 2022; Kirtana and Seetharaman, 2022). Epigenetics linked to MPs and NPs represent a research field of growing interest. Here, we collect published literature on the epigenetic alterations and gene expression modulation induced by MPs and NPs.

It has been widely documented that nanomaterials in general cause changes in gene expression in several exposed organisms, from microrganisms to plants, especially if the particles are charged or metal (Van Aken, 2015). For instance, Kaveh et al. (2013) demonstrated that exposure of *A. thaliana* to Ag nanoparticles increased plant growth at low doses and decreased plant growth at higher doses. Genes associated to metal and oxidative stress response were up-regulated while genes involved to pathogens and hormonal stimuli response were down-regulated. Changes in gene expression due to exposure to nanomaterials are also frequently reported in animal models where many biological processes are studied using molecular markers related to oxidative stress, cell metabolism, DNA repair and cell cycle regulation (Burkard et al., 2020).

## Plants

The impacts of pollutants on plants are particularly important since they are crucial components in the ecology of an environment (Barton et al., 2019; Yin et al., 2021). Frequently observed outcomes of the MPs and NPs exposure in plants are reduced nutrient uptake from soil and photosynthesis rate (Matthews et al., 2021; Karalija et al., 2022). MPs, according to their size, usually cannot be absorbed by plant root systems, but recent data show that they might enter plant tissues through stomata. Conversely, NPs can easily enter the plant root system and cases of transport through the xylem to the upper parts of the plant have been recorded (Karalija et al., 2022).

In addition to the toxicity mechanisms described before, it has been shown that MPs and NPs can also induce alterations in gene expression in various plants. Lagarde et al. (2016) showed that in the microalga *Chlamydomonas reinhardtii*, polypropylene and high-density polyethylene MPs of sizes 400 and 1,000 μm (100 mg in both conditions) tend to form alga-MPs aggregates. Since it is known that extracellular polysaccharides (EPS) provides an attractive force that enhance aggregation of cells to abiotic surfaces, the authors measured RNA levels of genes involved in sugar biosynthesis pathways, such as upregulation of UDP-glucuronate decarboxylase and UDP-glucose-4-epimerase involved in xylose biosynthesis and a reduction for UDP-glucose-4-6-dehydratase. The expression of these genes was studied by quantitative Real Time-PCR (qRT-PCR) and interestingly they found that polyethylene appears to over-express these genes more than polypropylene.

In *Allium cepa,* a reduction in mitotic rate was observed in root cells, due to a dose-dependent (100 nm nanopolystyrene in different concentrations 25, 50, 100, 200, and 400 mg/L) decrease in gene expression of the G2-M transition factor *cdc2* by qRT-PCR (Maity et al., 2020).

Sun et al. (2020) observed in *A. thaliana* that the administration of micro- and nanopolystyrene (less than 100 nm for NPs and between 0.1 and 5 mm for MPs) results in decreased root growth and elongation. A RNA-seq analysis showed that positive charged NP cause a more pronounced global gene expression alteration. More specifically, downregulation of genes involved in metabolic processes such as ROS, stimuli and stress responses was found for both charged NPs. A difference in upregulated gene expression was observed based on NPs charges.

The aforementioned results suggest that chemical composition of MPs and NPs could influence the biological effects and/or their intensity.

In wheat (*Triticum aestivum L*.)*,* Lian et al. (2022) used integrated differentially expressed gene analysis and weighted correlation network analysis (WGCNA) to analyse the molecular mechanisms of NPs phytotoxicity. They found that 100 nm NPs (concentrations of 0.01, 0.1, 1, and 10 mg/L) significantly altered carbon metabolism, aminoacid biosynthesis, mitogen-activated protein kinase (MAPK) signaling pathway, hormone signal transduction potentially leading to a reduction in biomass and compromising food yield and quality.

Nowadays, to our knowledge, well-defined epigenetics effects of MPs and NPs on plants are not reported in the scientific literature.

## Animals

Studies on epigenetic alterations in animals are more advanced than those in plants, but still very few in numbers. The standard organism used for pollutant toxicity studies is *Daphnia magna* (Lee et al., 2009; Pellegri et al., 2014).

Martins et al. (2018) demonstrated that exposure to 0.1 mg/L of 1–5 μm diameter polymer MPs from parental generation can impair survival of subsequent generations that may lead to an increased probability of extinction across generations. However, no molecular analyses have been carried out to determine possibly epigenetic causes, but the effects observed in the parental generation after exposure, which manifest themselves again in subsequent filial generations after a recovery period, in our opinion, support a possible epigenetic mechanism as a memory of parental exposure (Müller-Xing et al., 2014). If the observed effects on next generations, were caused by a DNA mutation, no recovery period would be expected. However this possibility cannot be ruled out.

Another validated bio-indicator of environmental stress are copepods being the main components of the freshwater interstitial communities (Di Cicco et al., 2021; Roncalli et al., 2021). In recent work by Lee et al. (2023), *P. nana* species were chronically exposed to 50 nm nanopolystyrene at a concentration of 10 mg/L. Multigenerational and transgenerational effects were evaluated in the study along with simultaneous presence of other environmental stresses such as water acidification (pH ranging from 7.0 to 8.0). The first result is that the co-presence of NPs and a second stress such as lower marine water pH led to a reduction in fertility in all exposed generations. Interestingly this fertility reduction was also observed in subsequent generations when only the parental generation was co-exposed. The authors, by whole-genome bisulfite sequencing, detected an increased DNA methylation in several genes (347 genes in the group in which each generation was exposed to NPs and 383 in the one where only the parental one was exposed) including those involved in responses to thermal stress, oxidative stress and cell death.

Among invertebrates, *Caenorhabditis elegans* is widely used to assess MPs and NPs toxicity. Yu et al. (2021) exposed the worm to 100 nm nanopolystyrene at a concentration of 50 mg/L for 72 h. In addition to numerous chromosomal aberrations during oocyte diakinesis and germline cell apoptosis, they observed transgenerational effects. Accordingly, significant levels of increased mRNA level of *ced-3,* −*4,* −*9,* genes involved in the process of apoptosis, were maintained for 4 generations after parental NPs exposure. The increased gene expression was associated to a DNA hypomethylation (by pyrosequencing) of proximal promoter of *ced-3* gene in all four generations. In the same study, by qRT-PCR analysis the researchers also observed a reduced transcription of epigenesis-related genes such as *met-2*, *set-2* and *spr-5*. They concluded that trans-generational effects is associated with epigenetic mechanisms*.* Wang et al (2021) exposed the worms to concentrations from 1 to 100 μg/L of 103 nm NPs and by qRT-PCR, observed reduced expression of methyltransferases *met-2* and *set-16*. The involvement of the epigenetic mechanism in the response of *C. elegans* to NPs (100 nm) was confirmed by Qu et al. (2019). In Hiseq 2000 sequencing, a deregulation of 37 intergenic lncRNAs were found and specifically a downregulation of *linc-50* and an increase in *linc-2, linc-9, linc-18* and *linc-61*. These lncRNAs are associated with a high number of biological processes such as development, immune system responses, cell proliferation. Li et al. (2020) using an intestinal specific mutant *mir-35* identified a signaling cascade of NDK-1-DAF-16/KSR-1/2 for the epigenetic control of toxicity response to nanopolystyrene (1, 10, 100, and 1000 μg/L of 100 nm). Moreover, other miRNAs, *mir-76, mir-38* and *mir-794,* have been linked in *C. elegans* to defects in homeostasis and gut response following nanopolystyrene exposure (Qiu Y. et al., 2020; Yang et al., 2020; Liu H. et al., 2021).

In *Drosophila melanogaster,* Zhang et al. (2020) studied the combination of MPs with the environmental contaminant Cadmium (Cd). Several combination of NPs and Cd were used: negative control group, MP(L) group (containing 200 μg/mL 1-μm MPs), MP(S) group (containing 200 μg/mL 0.1-μm MPs), Cd group (1.5 mM Cd), Cd + MP(L) group (containing 1.5 mM Cd and 200 μg/mL 1-μm MPs), and Cd + MP(S) group (containing 1.5 mM Cd and 200 μg/mL 0.1-μm MPs). Position-effect variegation (PEV) is a marker of gene silencing via heterochromatin spreading in *Drosophila* and it is caused from loss of epigenetic modifications, such as histone methylation and acetylation (Elgin and Reuter, 2013; Wang and Elgin, 2019). Cadmium alone induces gene silencing via PEV in somatic eye tissue. Although MPs alone does not induce PEV, in combination with Cd, PEV is enhanced. A mechanism involved in this effect could be DNA methylation as shown in Guan et al. (2019). To date, this work represents the first and unique that indicates a role of MPs to induce chromatin structure changes and therefore epigenetic alterations in fruit fly. With regard to gene expression, recent work (Matthews et al., 2021) shows that exposure to MPs and NPs of polystyrene does not alter the expression of genes involved in oxidative stress but results in a significant upregulation of the *hsp70* gene, which in *Drosophila* is induced by various physical, physiological and chemical stressors.

In *Danio rerio,* Qiang et al. (2020) to identify potential transgenerational effects on offspring generation, studied the expression of fish gonad-related genes. In particular, by qRT-PCR an increase in the mRNA expression of *hmgcra*, *hmgcrb* and *hsd3b2 genes* at 1,000 μg/L with 1 μm sized microplastics. Despite the changes in the expression of steroidogenic genes, they found no alteration in parental reproductive success and growth performance of offspring. Santos et al. (2022) observed a generalized reduction of survival and increased mortality after sub-chronic exposure to MPs and Cu alone and in combination. The authors investigated their impact on neuro-related genes and DNA methyltransferases and suggest that MPs and Cu, combined or not, can induce neurotoxicity by altering neural self-renewal, proliferation, differentiation and maturation related genes. Furthermore, they found reduced expression of *DNA methyl transferases*, known to be important factors in nerve development. Since the two classes of gene modulation cannot be directly linked going in opposite directions, the authors suggest the involvement of another molecular mechanism to be identified.

Pedersen et al. (2020) exposed fish to 10, 100, 1,000, and 10,000 ppb concentrations of 50 and 200 nm nanopolystyrene. They found significant hyperactivity compared to the negative control during hours of darkness associated with pathways related to nerve and movement diseases. In particular, by RNA Quant-Seq analysis differences were found in the expression of GABA channels as the likely cause of behavioral disorders. In addition, genes associated with lipid catabolism such as *ppar* and *aco* showed an initial increase with a subsequent decrease as concentrations rose (Cedervall et al., 2012).

Recently, interesting studies in mammals on transgenerational effects of MPs and NPs have been published. (Luo et al., 2019a; Luo et al., 2019b) investigated the effect of plastics during gestation and evaluated the potential effects on the mice offspring. A serum analysis of the metabolites showed an alteration of the molecules related to the metabolism of lipids and fatty acids together with a reduction in the weight of the offspring. Thus, using qRT-PCR, they found that genes regulating fatty acid transport (*Fabp1, Fatp2*), β-oxidation (*Ppar-α, Acox, Cpt1-α, Mcad*) and fatty acid synthesis (*Srebp1c, Fas, Acl, Scd1*) were significantly decreased in MPs-treated groups of F1 offspring (polystyrene MPs sizes 0.5 and 5 μm in diameter and 100 and 1,000 μg/L in drinking water). To evaluate the long-term consequences induced by MPs, the same authors analysed the transcriptome also in F2 generation. The effects found in F2 were much lower than those F1 offspring, although some genes were still significantly altered. These results show that there are still hidden molecular mechanisms that can cause adverse effects even in individuals not directly exposed through pathways yet to be determined.

In a recent study (Xiong et al., 2023), mice were exposed to 80 nm, 0.5 and 5 μm (100 mg/L) MPs and changes of renal fibrosis were also investigated. By RNA-Seq, about 70 genes associated with lipid storage, cellular response, and circadian regulation were differentially expressed in all sized MPs. In this work, the authors highlighted MPs as a potential important risk factor in kidney disease. Moreover, Thorson et al. (2021) studied in rats the transgenerational epigenetic effects induced by associated plastic compounds, including bisphenol A (BPA) di-(2-ethylhexyl) phthalate (DEHP), and dibutyl phthalate (DBP). They observed in males sperm changes in the methylation pattern, by Methylated DNA Immunoprecipitation-Seq Analysis, in genes associated with kidney and testicular diseases. Compounds were injected in different conditions: BPA 25 mg/kg BW/day, DEHP 375 mg/kg BW/day, and DBP 33 mg/kg BW/day) or vehicle control dimethyl sulfoxide (DMSO).

## Discussion

The widespread plastic waste emergency is now public knowledge. It is crucial to understand not only the general direct and immediate effects on living beings, but also the underlying molecular mechanisms and potential transgenerational implications. Here, we focused on epigenetics and gene expression modulation induced by MPs and NPs. To underline the importance of epigenetics responses, it is worth emphasising that epigenetics mechanisms are also associated with pathologies such as Alzheimer, Parkinson and cancer (Pavlou and Outeiro, 2017; Nebbioso et al., 2018; Migliore et al., 2022; Sahafnejad et al., 2023). Recent toxicogenomics investigations also discovered that epigenetic indicators of nanoparticles stress response were becoming more significant. For instance, it was seen that C2H2 zinc finger subfamily (C2H2-ZNF), that are central in regulating chromatin accessibility, is an evolutionary response shared by many organisms when exposed to nanomaterials to decrease their toxicity (Del Giudice et al., 2023). Considering that plastic fragments have now been found in various human districts such as lungs, placenta, and breast milk, this area of investigation assumes a key role (Ragusa et al., 2021; Jenner et al., 2022; Ragusa et al., 2022). Although few studies at present have addressed the epigenetics alterations of MPs and NPs (Lopez et al., 2022), it is becoming more evident the involvement of epigenetics on the effects observed in many model systems.

Among the mentioned papers in these review, it emerges alteration of epi-genes such as methyl-transferases and modulation of stress responses such as apoptosis and immune system. Between metabolic pathways, the lipid metabolism is affected in different animal organisms. For instance, the expression of the peroxisome proliferator-activated receptors *ppar* is altered both in *zebrafish* and mouse suggesting that metabolic energy imbalance is a common outcome of MPs and NPs exposure. Interestingly, the Shearwaters “plasticosis” was found similarly in mice indicating tissue fibrosis as another possible common outcome in vertebrates.

In recent years, there has been an improvement in sequencing techniques and bioinformatic tools/pipelines for analysing big “omics” data, but a challenge in plastics research area is the design of experiments. The environmental concentration, size and exposure pathways of MP and NPs have not been fully elucidated, so it is difficult to establish the best protocol to mimic real-life conditions and therefore extrapolate results for human health. Moreover, MPs have also been found in breast milk and placenta indicating that humans are continuously exposed even before their birth. From this perspective, the multi- and trans-generational studies are the most realistic approach.

Actions to clean up contaminated matrices and limit future plastic waste are crucial in addition to the research being done by the scientific community (Onyena et al., 2022).

Considering the ability of certain bacteria to bioremediate plastics, many studies have focused on exploiting these bacteria as a biotechnological opportunity for plastic polymer degradation and waste clean-up. (De Tender et al., 2017; Jacquin et al., 2019; Farda et al., 2022).

In conclusion, there is a need to better understand the toxicity mechanisms relating to MPs and NPs pollution, as well as to increase and improve useful technologies for cleaning environmental matrices, improve recycling laws, and most importantly, raise awareness among the general public worldwide, especially in developing nations (Meegoda and Hettiarachchi, 2023).
